# Iodine and bromine in fish consumed by indigenous peoples of the Russian Arctic

**DOI:** 10.1038/s41598-020-62242-1

**Published:** 2020-03-25

**Authors:** Nikita Sobolev, Andrey Aksenov, Tatiana Sorokina, Valery Chashchin, Dag G. Ellingsen, Evert Nieboer, Yulia Varakina, Elena Plakhina, Alexandra Onuchina, Magny Skinlo Thomassen, Yngvar Thomassen

**Affiliations:** 1 Northern (Arctic) Federal University named after M.V. Lomonosov, Arctic Biomonitoring Laboratory, Severnaya Dvina Emb. 17, 163002 Arkhangelsk, Russia; 2 Northwest Public Health Research Centre, 2-Sovetskaya str. 4, 191036 St. Petersburg, Russia; 3 National Institute of Occupational Health, P.O. Box 5330, Majorstua, N-0304 Oslo Norway; 4 Department of Biochemistry and Biomedical Sciences, McMaster University, Hamilton, ON Canada; 5 Norwegian University of Life Sciences, N-1432 Ås, Norway; 6 Institute of Ecology, National Research University Higher School of Economics, Myasnitskaya str. 20, 101000 Moscow, Russia

**Keywords:** Analytical chemistry, Environmental chemistry, Inorganic chemistry, Medicinal chemistry

## Abstract

Fish muscle may constitute one of the main sources of iodine (I) for the indigenous peoples of the Russian Arctic, although limited information is available about its content in commonly consumed fish species. In the current study, bromine (Br), I, the essential elements (copper, selenium and zinc) and other non-essential elements — specifically mercury, arsenic (As), cadmium, lead and nickel — have been quantified in 10 fish species consumed by people living in the Nenets and Chukotka Regions. Fish muscle was analysed by ICP-MS after nitric acid or tetramethylammonium hydroxide digestion. Certified reference materials were employed and concentrations are reported as geometric means (GMs). Atlantic cod (6.32 mg/kg) and navaga (0.934 mg/kg) contained substantially higher amounts of I than all other fish species, while broad whitefish had the lowest (0.033 mg/kg). By comparison, navaga contained more Br (14.5 mg/kg) than the other fish species, ranging 7.45 mg/kg in Atlantic cod to 2.39 mg/kg in northern pike. A significant inter-fish association between As and I in freshwater and marine fish was observed, suggesting common sources and perhaps parallel absorption patterns. Only Atlantic cod and, to lesser extent, navaga constituted significant dietary sources of I.

## Introduction

Fish foods provide humans with important nutrients as they contain high-quality proteins, marine n-3 fatty acids, vitamin D, vitamin B12, as well as the essential elements I, copper (Cu), selenium (Se) and zinc (Zn). A number of studies have shown that fish consumption is beneficial in preventing the development of cardiovascular disease and for fetal and neurological development. Insufficient I intake during pregnancy has been shown to cause deficiencies in maternal thyroid hormones (THs) that can result in impaired cognitive disorders in children. This specific impact is initiated during the first half of pregnancy when the fetus is entirely dependent on maternal THs^1^. Impairment of neurodevelopment has long been known in case of severe I deficiency (ID)^2^. Some more recent epidemiological studies suggest that mild maternal ID associates with delays in child cognitive development, lower verbal IQ and lower educational outcomes later in life^3,4^. Currently, I and iron (Fe) deficiencies remain two of the leading causes of preventable brain damage in fetuses, newborn and infants and ID as a cause of thyroid disorders in adults^2,5^.

Fish food in human nutrition may also be a source of environmental contaminants such as persistent organic pollutants (POPs) and mercury (Hg). Both have the potential to disturb the neurological development of children among other impacts, such as the perturbation of thyroid hormones [for a summary, see the 2015 Arctic Monitoring and Assessment Programme (AMAP) Assessment Report^6^]. Risk assessment of exposure in epidemiological studies to these environmental neuro-toxicants rarely takes the potential confounding of I status into consideration. Among others, the AMAP has been in effect for 3 decades and has focused on the influences of environmental pollutants on the health of individuals living in the Arctic regions of North America and Europe, especially among indigenous populations^6^. Somewhat surprisingly, neither the dietary intake of I nor its status has been assessed even though its deficiency has been known to exist among Russian Arctic populations^7,8^.

Considering the chemical similarity of I to Br, it is not surprising that in its ionic form (Br^−^) it may well be “an essential trace element for all animals since it appears to be required for the assembly of collagen IV scaffolds in tissue development and architecture”^9^. Rat experiments have shown that intake of Br^−^ in excess can replace I^−^ in tissues; decrease the latter’s accumulation in the thyroid and skin and increase its renal excretion; induce hypothyroxinemia after prolonged intake and is transferred through mother’s milk to the suckling^10^ and that its goitrogenic effects in rats are significant at moderate ID^11^. Furthermore, Molin *et al*.^12^ have shown that consumption of seafood rich in various organic As species was strongly associated with an increase of thyroid-stimulating hormone (TSH) levels in plasma. Indeed, a change in TSH was positively associated with the total As in plasma and this observation varied with the type of seafood ingested. These findings imply that organic compounds of As, which constitute the dominant form of this element in fish, may influence thyroid hormones and function.

As a component of a Russian mega-grant study to quantify the presence of POPs and Hg in food items consumed by pan-Russian Arctic indigenous populations, the amounts of essential and non-essential elements in fish species consumed were also quantified^13,14^. The latter included the non-metals As, Br, Se and I. Fish contain high amounts of As and I compared with other food items. In fact, some fish constitute an excellent source of I^15–17^. Its content varies considerably within and between species as does As. Thus, classification of fish populations by their As and I contents is difficult, as there are large locally-dependent variations between species and even within the same species^18^. Marine fish species low in fat appear to have the highest content of As and I and, in general, they have about six-fold higher I concentrations than freshwater fish^19,20^. Concentrations of I in fish also depend on the season and geographic location^21^. Even though the I contents are considerably lower than that of fish, milk and dairy products remain a primary source in many countries when I is added to fodder^22^.

There is very little information about the human daily nutritional intake of Br. For the central regions of the European part of Russia, daily consumptions (dependent on the type of diet) ranging 5.54 to 11 mg have been reported^23^. Comparable daily averages have been established, specifically: 3.6 mg in the UK^24^; and 2.9 mg for Ukrainian males and 10.5 mg in Japanese males^25^.

To the best of our knowledge, only Gorbunov *et al*.^23^ have reported the amounts of Br in the fish and shellfish consumed by pan-Russian Arctic indigenous populations. Despite the fact they have measured the Br content in a large number of fish species, only wet weight average concentrations (in mg/kg) were published: river fish (2.2 ± 1.6); salt-water fish (12.9 ± 8.8); shrimps (350 ± 227); and squid (24.4 ± 9.3). Previously we have reported the concentrations of selected toxic (As, Cd, Hg, Pb, Ni) and essential (Co, Cu, Se, Zn) elements measured in 7 fish species consumed by the indigenous peoples in the Nenets Autonomous Region within the Barents Sea region of the north-eastern part of European Russia^13,14^.

The primary objective of the present study was to quantify Br and I in 10 fish species regularly consumed by people living in the Russian Arctic, as well as of 4 essential and 5 toxic inorganic elements in 3 fish not previously reported on.

## Results

**Figure 1:**
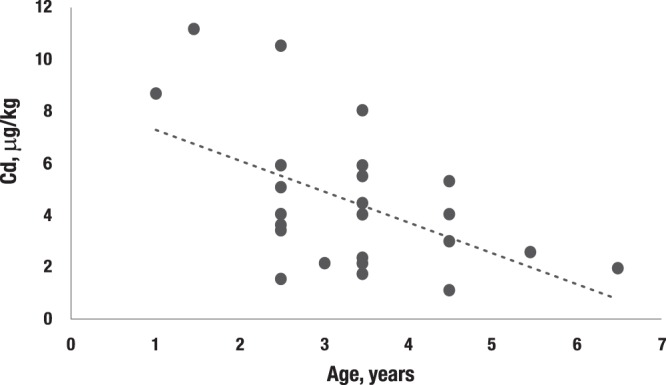
Decrease of Cd in Atlantic cod muscle with age.

We have previously published the concentrations of selected essential and toxic elements (namely, Cu, Co, Se, Zn, Ni, Hg, Pb, Cd, As) in muscle tissues of fish species collected in the Nenets Region of the European Russian Arctic^13,14^. The current study is an extension of these efforts. Findings for Atlantic cod consumed by the Nenets residing on Kolguyev Island and in pink salmon and broad whitefish consumed by the Chukchi peoples of Chukotka are hereby added to our database^14^. Pertinent concentration data for the indicated elements are summarized in Table 1. In general, the observed concentrations of the three additional fish species are comparable to those published earlier for 8 fish species, with As and Cd the exceptions. The observed As concentrations are considerably higher, and it is noteworthy that more than a 20-fold difference existed between the minimum and maximum concentration for Atlantic cod. On average, it contained a fourfold higher concentration of Cd than in pink salmon and broad whitefish. For Atlantic cod, there was a decrease in the muscle Cd concentration with age (p = 0.007, with Pearson r = −0.52; Fig. 1). By contrast, an apparent increase (not shown) with age of Hg was also evident (p = 0.027, r = 0.44).

**Table 1:** Geometric means (GM) and ranges of wet weight concentrations of elements in muscle of different fish species consumed by Russian Arctic indigenous peoples.

Fish details and elements measured	Atlantic cod-Barents *(Gadus morhua)* (N = 25)		Pink salmon-Chukotka (*Oncorhynchus gorbuscha*) (N = 5)		Broad whitefish-Chukotka *(Coregonus nasus)* (N = 5)	
	GM	Min-Max	GM	Min-Max	GM	Min-Max
Age (year)	3.2	1–6.5	1+*		5.0	4.5–5.5
Weight (kg)	0.64	0.26–1.05	0.57	0.50–0.65	1.22	1.11–1.35
Hg (µg/kg)	57	48–74	71	65–73	72	62–80
As (µg/kg)	17800	3500–73500	308	252–454	19	13–27
Se (µg/kg)	314	246–446	376	320–428	228	171–279
Cd (µg/kg)	3.9	1.0–11.0	1.5	1.2–1.8	1.1	0.7–1.8
Pb (µg/kg)	3.8	0.88–8.9	2.3	1.3–6.1	3.2	2.1–6.5
Co (µg/kg)	3.0	1.3–12.0	2.3	1.2–5.1	10.4	7.0–18.3
Ni (µg/kg)	27	17–66.8	21	12–31	11.5	9.2–17.2
Cu (µg/kg)	259	158–404	467	410–576	328	223–404
Zn (mg/kg)	5.13	3.94–6.29	4.62	4.42–4.90	4.03	3.71–4.76

Abbreviations: N - Number of fish; *Age of all fish exceeded 1 year.

**Figure 2:**
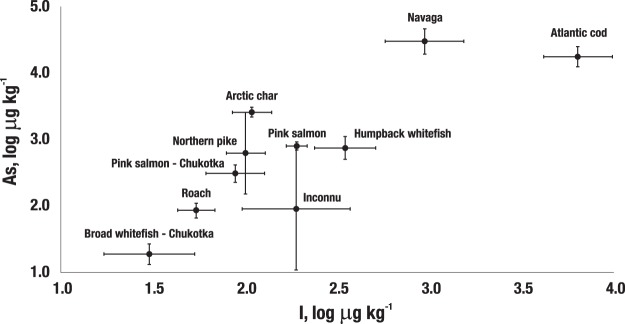
Geometric mean concentrations (log-scale) of As and I with 95% confidence intervals in 10 fish species consumed by indigenous people of the Russian Arctic.

The muscle concentrations of I and Br in all nine fish species tested are summarized in Table 2. On average, Atlantic cod contained substantially higher amounts of I than all other fish species (6.32 mg/kg), and there was a 65-fold difference between the minimum and maximum values. The other cod fish, namely navaga *(Eleginus nawaga)*, also featured significantly more I (0.934 mg/kg) than the other fish species tested. For example, concentrations ranged from 0.395 in humpback whitefish to as low as 0.032 mg/kg in broad whitefish caught in Chukotka. For Atlantic cod, there was a decrease in the muscle log I concentration with age (p = 0.03, with r = −0.43). For Br, the situation differed, specifically: navaga contained significantly more Br (14.5 mg/kg) compared to the other fish species that ranged from 7.45 in Atlantic cod to 2.39 mg/kg in northern pike. It should also be noted that the ratio of Br and I concentrations in the different fish species varied considerably, namely from 1.2 in Atlantic cod to 95 in broad whitefish from Chukotka. Not surprisingly, there were significant associations (log scale) between the concentrations of I and Br in all fish (n = 103, p = <0.001; r = 0.49); as well as of As and I (n = 103, p = <0.001, r = 0.77) and of As and Br (n = 103, p = <0.001, r = 0.65). In Fig. 2, the GMs (log scale) with 95% confidence intervals of the concentrations of As and I in the various fish species are depicted. Generally speaking, a positive relationship is evident, although the inconnu findings appear to be somewhat of an outlier.

**Table 2:** Geometric means (GM) and range of wet weight concentrations of bromine (Br) and iodine (I) in muscle of different fish species.

Fish species	Age (year)		Weight (kg)		Br (mg/kg)		I (mg/kg)		Ratio Br/I
	GM	Min-Max	GM	Min-Max	GM	Min-Max	GM	Min-Max	
Arctic char^a^ *(Salvelinus alpinus)* (N = 11)	4.2	2.5–6.5	0.65	0.26–1.05	5.63	3.68–8.02	0.108	0.066–0.259	52
Pink salmon^a^ *(Oncorhynchus gorbuscha*) (N = 12)	1+*		1.04	0.81–1.64	5.12	4.47–6.02	0.190	0.132–0.243	27
Navaga^b^ *(Eleginus nawaga) (N = 10)*	4.4	3.0–6.5	0.21	0.13–0.38	14.5	8.8–24.7	0.934	0.268–2.84	16
Humpback whitefish^c^ *(Coregonus pidschian)* (N = 12)	7.0	5.0–10.0	0.44	0.38–0.57	6.47	5.34–8.68	0.346	0.086–0,711	19
Northern pike^d^ * (Esox lucius)* (N = 7)	6.6	3.5–0.5	2.30	0.81–5.62	2.39	1.86–3.23	0.100	0.071–0.138	24
Roach Indiga^d^ *(Rutilus rutilus) (N = 10)*	10.4	8.5–3.0	0.31	0.26–0.38	2.54	1.73–3.57	0.055	0.040–0.115	46
Inconnu^c^ *(Stenodus nelma) (N = 6)*	8.0	5.0–12.5	1.11	0.42–2.30	4.32	2.06–9.92	0.188	0.056–0.347	23
Atlantic cod^b^ *(Gadus morhua)* (N = 25)	3.2	1–6.5	0.64	0.30–2.53	7.45	5.39–13.4	6.32	0.514–33.6	1.2
Pink salmon-Chukotka^a^ (*Oncorhynchus gorbuscha*) (N = 5)	1+	1–2	0.57	0.50–0.65	5.34	4.30–6.17	0.088	0.066–0.144	61
Broad whitefish-Chukotka^c^ *(Coregonus nasus) (N = 5)*	5.0	4.5–5.5	1.22	1.11–1.35	2.85	1.73–24.7	0.033	0.018–0.055	95

Abbreviations: N - Number of fish; ^a^Anadromous fish. ^b^Seawater fish. ^c^Semi-anadromous fish. ^d^Freshwater fish. *Age of all fish exceeded 1 year.

## Discussion

Iodine concentrations in Atlantic cod (Table 2) were significantly higher than for other species with a GM of 6.32 mg/kg (ww) in muscle. High I content in Atlantic cod have been observed previously, such as a mean of 2.5 mg/kg (ww) in Barents Sea cod both in 2001 and 2018^21,26^. By comparison, Atlantic cod from the Norwegian Sea had a mean of 4.0 mg/kg and 0.96 mg/kg for those from the North Sea. A large variation between individuals was observed in both studies, which is also evident in the present work (specifically, 0.514–33.6 mg/kg). While Atlantic cod have been extensively studied, information on I contents is limited for the other species included in our study. In the Norwegian Food Composition Database^16^, I contents of 0.2 mg/kg and of 0.12 mg/kg in Arctic char muscle are stated, which are comparable to our mean value of 0.108 mg/kg.

Inconnu, a whitefish in the Salmonidae family, and the humpback whitefish are both anadromous. This means that they not only spend part of their existence in the ocean but also in fresh water. Humpback whitefish is known to rear in channel ponds, sloughs, estuaries and marine environment until they are mature. The roach, northern pike and broad whitefish are freshwater species, although the broad whitefish may move among freshwater, brackish or marine water to feed and overwinter. For freshwater fish, the I contents have generally been recognized as significantly lower than of seawater species^19,27^. This is confirmed in the current study, as the freshwater species had low I contents.

Iodine can be taken up by fish from the water, by way of the gills as well as from their diet. However, its absorption is dependent on the calcium water concentrations^21,28^. Freshwater species depend more on dietary sources of I than marine species. Since seawater contains more I than freshwater and since marine plants and marine prey are rich sources of I, higher amounts of this element are found in marine species especially in lean fish^26,29^ in comparison to fatty marine fish like halibut, mackerel, sardine and herring. The reasons for this is still unknown^26,30^. The differences seen between and within species may thus be explained by differing diets, environments, ages and sizes.

The diet of Atlantic cod living in the Barents Sea has been studied for many years in collaborative studies between Russian and Norwegian scientists and authorities^31^. For small fish, less than 20 cm in length, euphausiid crustaceans constitute a primary component of their food. On maturing, more and more of the Atlantic cod diet consists of fish including cod and other species. Actually, at a size of 5 kg and above, more than 70% of the diet seemed to be smaller cod. This should perhaps lead to an accumulation of I in the bigger/older cod. However, this was not evident in our study. For our limited data set of cod species with an average age of 3.2 y, there was a significant decrease of the I content with age.

The Br contents found in the fish species studied were, as expected, higher than for I (see Table 2). A slight inter-fish species association between Br and I contents is present (n = 103, p = <0.001, r = 0.65), while only a significant intra-fish species association was registered for Atlantic cod (n = 25, p = 0.033, r = 0.43). However, the Br/I ratio varied substantially in our study, specifically from 1.2 in Atlantic cod to 95 found in the broad whitefish from Chukotka. A similar high variation in this ratio, from 6 in mackerel (*Scomber scombrus*) to 112 in sardines (*Sardina pilchardus*), has previously been reported^32^. Since I is needed for production of thyroid hormones, a daily intake from 70 µg/day for children (7–11 months) and up to 150 µg/day for individuals older than 18 years have been recommended^33^. According to Montag *et al*.^32^, studies using ^82^Br have not shown any incorporation of Br in thyroxin, while Clode *et al*. in 1962^34^ and based on rat studies have suggested that increased levels of Br could inhibit the synthesis of thyroxin. Excess intake of Br can cause goitrogenic effects in rats, which are rendered significant at moderate I deficiency^11^.

The I and Br contents in fish in relation to intake among indigenous peoples of the Russian Arctic was the primary focus of the present study. All the fish species included are frequently consumed by them, and thus should be considered as a nutritional source of the element I. Due to their I content (relatively speaking), only Atlantic cod and to lesser extent navaga constitute significant sources of it for dietary intake. Considering that the mean concentration of 6.32 mg/ kg muscle wet weight was found in the Atlantic cod, a 100 g cutlet/week would provide about 2/3 of the 150 micrograms of I per day recommended by the European Food Safety Authority (EFSA)^33^; for navaga, a 7-fold larger intake is required. While an adequate intake of I is essential for humans, to our knowledge no comparable requirement exists for Br. However, McCall *et al*. have proposed that Br is an essential element and is required for the formation of the sulfilimine bond (−S = N−) within collagen IV^9^. Consequently, a deficiency of Br intake may well have implications for human health and disease.

When compared with our previous findings for the Nenets Autonomous Region^13,14^ and with the exception of As, the observed concentrations of the elements in the three additional fish species examined were comparable. Arsenic in Atlantic cod ranged from 3500 to 73500 µg/kg (ww) and a GM of 17800 µg/kg (ww). By comparison, only a value of 19 µg/kg (ww) was observed for the broad whitefish from Chukotka. Generally, marine species contain higher amounts of As than freshwater fish, and Atlantic cod is known to have especially high amounts. Sloth *et. al*.^35^ have reported As concentrations in the 15–17 mg/kg range, which are comparable to the values observed in the current study. The Cd concentration of 3.9 µg/kg found in Atlantic cod muscle is also in agreement with the values we reported earlier for Arctic char, navaga and pink salmon. However, samples of the latter from Chukotka featured considerably lower Cd concentrations in muscle tissues (specifically, 1.5 µg/kg) than pink salmon caught in the Nenets region that spend most of their lives in sea-water habitats. As previously suggested for Barents Sea cod, the significant decrease in Atlantic cod Cd with fish age is most likely related to the gradual change in diet, from amphipods, euphausiids and copepods rich in Cd to mostly smaller fish^31,36^.

Our observation of a significant inter-fish association between As and I in fresh water and marine fish has, to the best of our knowledge, not been described previously. This suggests that, generally speaking, there may be common sources of both elements and a parallel absorption patterns even though there is a large difference in their chemistry in water environments (i.e., coastal zones, rivers, estuaries and lakes).

## Materials and Methods

### Sample collection and preparation

**Figure 3:**
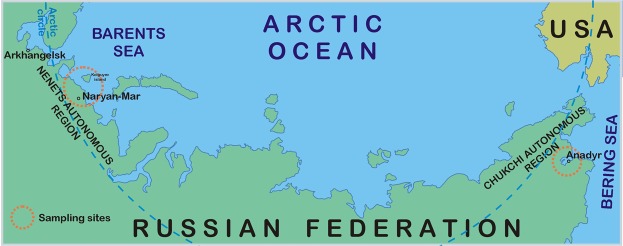
Map showing the sampling sites. The map was created using CorelDRAW Graphics Suite X4 software (education license), license certificate № 30064931. (https://www.coreldraw.com/); the topographic base of the map was created with Natural Earth Free Vector and Raster Map Data (https://www.naturalearthdata.com). (Map: Andrey Aksenov).

Detailed information about the selection and field sampling of the fish species in the Barents Region are outlined by Sobolev *et al*.^13,14^. In addition to the previously collected and analysed fish, Atlantic cod (Gadus morhua) was purchased from local fishermen in the Bugrino village on Kolguyev Island, which is located in the south-eastern Barents Sea as shown in Fig. 3. Furthermore, pink salmon (Oncorhynchus gorbuscha) and broad whitefish (Coregonus nasus) were collected in indigenous communities residing in the Chukotka Peninsula of North-Eastern Russia (see Fig. 3).

Due to the absence of any freshwater habitat on Kolguyev Island, Atlantic cod is the main marine fish species consumed by the local Nenets there. The latter constitute the majority of the islandic population and who depend on fishing, reindeer farming and trapping. The Chukotka collection sites were on the shores of the Chukchi Sea and in the Bering Sea region of the Arctic Ocean (see Fig. 3). The Barents Sea fish were obtained from May 2017 to July 2018. More specifically, the three supplementary fish species were caught in July 2018 (Atlantic cod) and pink salmon and broad whitefish in August 2018.

Whole fish were wrapped in food polyethylene film and placed in zip-lock sealed polyethylene bags and stored at −20 °C prior to transportation to the Arctic Biomonitoring Laboratory of the Northern (Arctic) Federal University in Arkhangelsk. The fish were subsequently thawed weighed, and their ages were determined by measuring the growth patterns in the otoliths (ear stones) located behind the brain in bony fish. This involved counting the number of opaque zones (annuli) from the primordium to the otolith edge^37^. Muscle tissue was separated from the carcass and then homogenized before further storage at −80 °C, and prior to analysis it was lyophilized.

### Elemental measurements

The elements As, Cd, Co, Cu, Hg, Ni, Pb, Se and Zn were quantified as described previously^13,14^. In short, portions of 0.25 g of lyophilized muscle were digested in 5 mL of ultrapure nitric acid and 5 internal standards were added before dilution to 25 mL. Quantification of the elements involved the use of an Aurora Elite inductively coupled plasma mass spectrometer (ICP-MS; Bruker Daltonik GmbH, Bremen, Germany), and the accuracy of the measurements was assured by the use of international certified materials and in-house quality controls. The day-to-day coefficient of variation for the in-house standards ranged 3.4–11.9%, while the recoveries for the elements in the reference materials bracketed 86–110% of the certified values.

For the measurement of I and Br, portions of 0.20 g of lyophilized fish muscles were weighed with an accuracy of ± 0.001 g into 100 ml modified polytetrafluoroethylene vessels. Subsequently 1 mL of 25% tetramethylammonium hydroxide (TMAH for use in syntheses, Merck KGaA, Darmstadt, Germany), 5 ml of ultrapure water and 0.25 ml of 1 mg/L tellurium (Te) internal standard solution (Spectrapure Standards AS, Oslo, Norway) were added. Samples were then digested in an MDS-6G (SMART) closed microwave digestion system (Shanghai Sineo Microwave Chemistry Technology Co., China) using the temperature program summarized in Table 3.

**Table 3:** Temperature program for the microwave digestion of fish samples.

Step #	Temperature, °C	Time, min	Power, W
1	130	10	400
2	160	5	400
3	190	15	400

After cooling to room temperature, the solutions were transferred to 50 mL polypropylene tubes (PP) (Sarstedt AG & Co. KG; Nümbrecht, Germany) and diluted to 25 mL with ultrapure water. The measurements of I and Br were carried out employing the Aurora Elite ICP-MS instrument equipped with a collision reaction interface (CRI) for reducing isobaric mass interferences. A concentric glass nebulizer and a Peltier cooled Scott double-pass spray chamber were used. The ^127^I isotope with ^125^Te used as internal standard was measured in both the no gas and gas modes. To reduce the polyatomic interferences of ^40^Ar^40^ArH^+^ with ^81^Br^+^, the CRI required a hydrogen-gas flow rate of 115 mL/min in the skimmer cone. TMAH matrix-matched calibration solutions were prepared daily in PP volumetric flasks from the certified primary aqueous stock solutions (I and Br, 1 mg/mL; Spectrapure Standards AS, Oslo, Norway). The moisture content of each sample was measured during the freeze-drying step. This allowed the recalculation of the individual moisture contents and to express the elemental concentrations in units of both mg/kg or μg/kg wet weight (ww)^14^.

### Quality assurance

The digestion vessels were cleaned between each sample digestion with nitric acid and TMAH, and this was followed by water rinsing. This and the preventive measures described above assured that blank sample signals were as clean as possible, thereby permitting suitable limit of quantifications (LOQ). The latter were established daily using 5 blanks and yielded typical values of 10 (I) and 110 (Br) µg/kg (ww). The accuracy of the measurements was established using relevant certified reference materials (CRM), specifically ERM-BB422 fish muscle (European Commission-Joint Research Centre, Institute for Research Materials and Measurements, Geel, Belgium)^38^ for I and IAEA-436 tuna-fish flesh homogenate (International Atomic Energy Agency, Vienna, Austria)^39^ for Br. For method development and daily quality assurance, commercially available lyophilized North Atlantic Ocean white fish powder (Seagarden, Nesttun, Norway) was analysed subsequent to each 10 fish samples^14^. The day-to-day coefficients of variation for this in-house quality control material and of observed recoveries are summarized in Table 4. Both ERM-BB422 and IAEA-436, as well as and Dolt-5 dogfish liver in addition to the in-house fish powder standard were employed to assess the quality of the measurements of other elements. The recoveries for the different elements ranged from 86 to 110% of the certified values (see ref. ^14^).

**Table 4:** Observed day-to-day recoveries and variability.

Element	Certified value ERM-BB422 (I) IAEA-436 (Br) µg^−1^g n = 5	Found, µg^−1^g	Recovery in %	In-house quality control material Whole fish powder µg^−1^g n = 5	Day-to-day variability of the in-house quality control material (RSD), %
I	1.4 ± 0.4	1.40 ± 0.02	100	8.9 ± 0.1	1.5
Br	14.8 ± 1.5	15.8 ± 1.2	107	50.5 ± 4.5	8.9

### Statistical analysis

The distribution of the variables was visually assessed and the associated skewness was calculated. Variables were log-transformed when the skewness exceeded 2.0. Average concentrations are presented as GMs. Univariate associations were evaluated using least square regression analyses that yielded the Pearson’s correlation coefficient (r) as the measure of association. Two-tailed p-values (p) <0.05 were considered to be of statistical significance. We have used the same statistical analysis in our previously published work reporting essential and non-essential elements in fish consumed by indigenous peoples of the European Russian Arctic^14^. The statistical package SPSS, version 25.0 (IBM Corp., Armunk, NY, USA) was employed for the statistical calculations.

## Data Availability

Any data and materials used in the current study are available from the corresponding author on reasonable request. The dataset that was generated as a result of present study can be downloaded from the open data repository^40^ 10.17632/schjsjfn3x.2.
